# Intraoperative PaO_2 _is not related to the development of surgical site infections after major cardiac surgery

**DOI:** 10.1186/1749-8090-6-4

**Published:** 2011-01-11

**Authors:** Juan Bustamante, Eduardo Tamayo, Francisco Javier Álvarez, Israel García-Cuenca, Santiago Flórez, Inma Fierro, José Ignacio Gómez-Herreras

**Affiliations:** 1 Departament of Cardiovascular Surgery. Hospital Universitario La Princesa. C/Diego de León 62. 28006. Madrid. Spain; 2 Department of Anaesthesiology and Reanimation. Hospital Clínico Universitario de Valladolid. Avenida Ramón y Cajal 3. 47005. Valladolid. Spain; 3 Department of Pharmacology and Therapeutics. Facultad de Medicina. Universidad de Valladolid. Avenida Ramón y Cajal 3. 47005. Valladolid. Spain; 4 Department of Anaesthesiology and Reanimation. Hospital Universitario Rio Hortega. Calle Dulzaina s/n. 47012. Valladolid. Spain; 5 Departament of Cardiac Surgery. Hospital Clínico Universitario de Valladolid. Avenida Ramón y Cajal 3. 47005. Valladolid. Spain

## Abstract

**Background:**

The perioperative use of high inspired oxygen fraction (FIO_2_) for preventing surgical site infections (SSIs) has demonstrated a reduction in their incidence in some types of surgery however there exist some discrepancies in this respect. The aim of this study was to analyze the relationship between PaO_2 _values and SSIs in cardiac patients.

**Methods:**

We designed a prospective study in which 1,024 patients undergoing cardiac surgery were analyzed.

**Results:**

SSIs were observed in 5.3% of patients. There was not significant difference in mortality at 30 days between patients with and without SSIs. In the uni and multivariate analysis no differences in function of the inspired oxygen fraction administrated were observed.

**Conclusions:**

We observed that the PaO_2 _in adult cardiac surgery patients was not related to SSI rate.

## 

Dear Editor,

The potential clinical benefits of the perioperative use of high inspired oxygen fraction (FIO_2_) for preventing surgical site infections (SSIs) have attracted great interest in recent years. Trials by Greif et al. [1] and Belda et al. [2] demonstrated that SSIs decreased significantly following colon surgery in patients who received 80% oxygen intraoperatively and for the first hours following surgery.

In the sphere of cardiac surgery, SSIs are serious complications associated with extended hospital stay, increased hospital costs, and higher mortality and morbidity rates [3]. Thus, in 2005 our Department of Anesthesiology and Reanimation adopted a clinical strategy of administering 50% oxygen without nitrous oxide during anesthesia and for the first 6 postoperative hours in an effort to decrease SSIs.

In contrast to the findings of Belda et al. [2], clinical trials by Pryor et al. [4] and, more recently, by Meyhoff et al. [5], found no difference in SSI risk when 80% oxygen rather than 30% oxygen was administered during abdominal surgery and for 2 hours postoperatively. Their findings suggested that perioperative hyperoxia was not effective in reducing SSIs. These reports add to the evidence base surrounding the potential role of high FIO_2 _in SSI prevention.

The rationale for administering high FIO_2 _to prevent SSIs is to produce a high PaO_2 _and thereby increase the PsqO_2 _(tissue oxygen partial pressure), since oxidative killing by neutrophils is the primary defense against surgical pathogens. The risk of infection is thus inversely related to PsqO_2 _[3]. Our aim in this study was to analyze the relationship between PaO_2 _values and SSIs.

We designed a prospective study that analyzed the data from 1,024 consecutive patients who underwent cardiac surgery with extracorporeal circulation at our institution from January 30, 2007 to June 30, 2009. Transplant patients were excluded. The patients were categorized according to the presence or absence of SSIs. The study was approved by the hospital's Research Commission, and all participants provided informed written consent. The Center for Disease Control and Prevention (CDC) criteria [6] were used to define SSIs. The SPSS software package (version 15) was used for statistical analysis. A *p *≤ 0.05 was considered significant.

To assess risk factors for SSI, we used one-way analysis of variance for univariate continuous variables and the chi-square test for categorical variables. In addition, we conducted Fisher's exact test whenever the chi-square expected value of at least one cell was less than 5.

We avoided multicollinearity among the explanatory variables by performing collinearity diagnostic analyses. We performed the stepwise selection of variables from the models with the following criteria: Tolerance greater than 0.4 or variance inflation less than 2.5, condition number less than 10, and a variance of two or more variables no greater than 0.5.

SSIs developed after cardiac surgery in 54 (5.3%) patients, 28 (2.8%) superficial or deep incision SSIs and 26 (2.5%) organ/space SSIs. The intraoperative and postoperative PaO_2 _values were not associated with an increased risk of SSI either by univariate or multivariate analysis (Table 1). The 30-day mortality rate was similar in both groups: patients without SSIs, n = 72 (7.4%) vs. patients with SSIs, n = 4 (7.4%); (*P *= .11).

**Table 1:** Characteristics and preoperative, intraoperative, and postoperative data for patients with and without surgical site infections (SSIs).

Characteristics	Patients Without SSI (n = 970)	Patients With SSI (n = 54)	Univariate OR (95% CI)	*P *value	Adjusted OR **(95% CI)**^**b**^	*P *value
**Preoperative value**						

Age, mean (SD), years	68.2 ± 10,1	69.07 ± 10,9	1.009 (0.981 to 1.03)	0.54		

Sex, male/female	591 (60.9)/379 (39.1)	37 (68.5)/17 (31.5)	1.396 (0.77 to 2.51)	0.26		

Underlying conditions, No. (%)						

Diabetes mellitus	285 (29.4)	16 (29.6)	1.01 (0.55 to 1.84)	0.97		

Hypertension	427 (44)	27 (50)	1.27 (0.73 to 2.28)	0.39		

Chronic renal failure	50 (5.2)	2 (3.7)	0.70 (0.16 to 2.98)	0.64		

Chronic obstructive pulmonary disease	202 (20.8)	18 (33.3)	1.90 (1.05 to 3.41)	**0.03**		

Peripheral vascular disease^a^	74 (7.6)	2 (3.7)		0.28		

Additional drugs, No (%)						

β-blockers^a^	435 (44.9)	21 (38.9)	1.28 (0.72 to2.27)	0.39		

Statin	373 (38.5)	23 (42.6)	0.84 (0.48 to 1.47)	0.55	1.29(0.71 to2.33)	0.39

Corticosteroids	19 (2.0)	1 (1.9)	0.94 (0.12 to 7.19)	0.95		

**Intraoperative values**						

Antibiotic prophylaxis, No. (%)						

Cefazolin	938 (96.7)	46 (85.2)	0.19 (0.008 to 0.44)	**0.001**	4.90(2.07 to11.61)	**0.0001**

Teicoplanin	32 (3.3)	8 (14.8)		**0.001**		

Surgical procedure, No. (%)						

Valve	490 (50.5)	31 (57.4)	1.33 (0.76 to2.32)	0.32		

CABG	296 (30.5)	14 (25.9)	0.8 (0.42 to 1.49)	0.47		

Valvular + CABG	184 (19.0)	9 (16.7)	0.85 (0.41 to 1.78)	0.67		

Total CPB time, mean (SD), min	92.8 ± 38.2	96.3 ± 35.7	1.002 (0.99 to 1.009)	0.502	1.001(0.99 to1.009)	0.77

Aortic cross-clamp time, mean (SD), min^a^	66.7 ± 29.04	69.5 ± 26.6	1.003 (0.99 to 1.01)	0.48		

Glucose, mean (SD), mg/dL^a^	180.2 ± 51.4	178.5 ± 48.5	0.99 (0.98 to 1.001)	0.07	1.00(0.99 to1.01)	0.95

PaO_2_, mean (SD), mm Hg^a^	148.4 ± 38.4	150.1 ± 34.2	1.001 (0.99 to 1.008)	0.74		

Hematocric during CPB, mean (SD), (%)	26.5 ± 4.4	25.8 ± 3.7		0.25		

**Postoperative**						

Duration of mechanical ventilation, mean (SD), days	51.4 ± 200.7	44.5 ± 146.3		0.805		

Glucose, mean, mg/dL 1-h ICU admission	166.2 ± 47.5	159.6 ± 52.4	1.001 (0.99 to 1.008)	0.32	0.99(0.98 to1.01)	0.19

8-h ICU post-admission^a^	169.1 ± 63.02	156.30 ± 40.8	0.996 (0.98 to 1.003)	0.14		

Core temperature, ICU admission, mean,°C	36.1 ± 0.7	36.1 ± 0.6	1.152 (0.78 to 1.696)	0.47	1,13(0.74 to1.71)	0.56

PaO_2_, mean (SD), mm Hg 1-h ICU post-admission	134.8 ± 41.3	136.5 ± 39.5		0.77	1.00(0.99 to1.01)	0.29

8-h ICU post-admission	130.1 ± 37.5	124.4 ± 34.02		0.27	0.99(0.98 to1.00)	0.22

Leukocyte, ICU admission, mean (SD),mm^3^	10934.5 ± 3826.5	11316.4 ± 3611.01	1.000 (1.000 to1.000)	0.47		

Hematocric, ICU admission, mean (SD), (%)	30.3 ± 4.7	31.5 ± 4.0	1.06 (0.99 to 1.12)	0.06		

Units red-cell transfusion, mean (SD)	2.02 ± 2.8	2.2 ± 2.5	1,027 (0.94 to 1.121)	0.54		

Mediastinal bleeding, mean (SD), mm^3^	828.9 ± 554.3	709.9 ± 92.5	1.000 (0.99 to 1.000)	**0.03**		

Complications, No. (%)						

Cardiac	72 (7.4)	6 (11.1)	1.5 (0.64 to 3.75)	0.32		

Respiratory failure	89 (9.2)	3 (5.7)	0.59 (0.18 to 1.93)	0.38		

Stroke	20 (2.1)	2 (3.7)	1.82 (0.41 to 8.0)	0.42		

Acute renal failure	61 (6.3)	8 (14.8)	2.63 (1.17 to 5.88)	**0.01**		

Length of stay, mean (SD), days						

Preoperative^a^	10.4 ± 9.8	12.1 ± 8.8	1.01 (0.99 to1.03)	0.209		

In the ICU stay after surgery^a^	4.4 ± 9.4	4.1 ± 6.6	0.99 (0.96 to 1.03)	0.81		

Postoperative^a^	13.8 ± 17.9	35.6 ± 19.5	1.03 (1.02 to1.04)	**0.0001**		

In the hospital	24.2 ± 20.2	47.8 ± 20.3	1.03 (1.01 to 1.04)	**0.0001**	1.01(1.008 to1.02)	**0.0001**

Mortality, No. (%)^c^						

In-hospital	76 (7.8)	7 (13.0)		0.17		

30 days	72 (7.4)	4 (7.4)	0.99 (0.35 to2.85)	0.99		

90 days	73 (7.5)	6 (11.1)	1.53 (0.63 to 3.70)	0.34		

Abbreviations: SD, standard deviation; SSIs, surgical site infections; PaO_2_, partial pressure of oxygen; CI, confidence interval; ICU, intensive care unit; OR, odds ratio; CABG, coronary artery bypass graft; CPB, cardiopulmonary bypass.

Our results agree with the results of the trials conducted by Pryor et al. [4] and Meyhoff et al. [5] in that perioperative hyperoxia was not effective in reducing SSIs. PsqO_2 _is typically lower than the PaO_2 _level by a factor of two to four. As might be expected, tissue oxygenation improves much less than arterial oxygen in response to supplemental oxygen administration. Sternal wound oxygenation increased by an average of 4 mm Hg (from 23 to 27 mm Hg) with supplemental oxygen at 50% [3].

The data from prior studies [4,5], as well as the present results, leads us to question our policy to routinely administer a high inspired oxygen fraction to cardiac surgery patients in order to prevent SSIs. In summary, the PaO_2 _in adult cardiac surgery patients is not related to SSI rate. The strategy of administering supplemental inspired oxygen to reduce the incidence of SSIs does not appear to be clinically useful.

## Competing interests

The authors declare that they have no competing interests.

## Authors' contributions

JB and ET had full access to all of the study data and takes responsibility for the integrity of the data and the accuracy of the data analysis. Both authors contributed equally to the study. Study concept and design: ET, JB, FJA, IGC, JIGH. Data acquisition: JB, ET, FJA, IGC, IF, JIGH. Analysis and interpretation of data: ET, IF, SF, FJA, IGC, JB, JIGH. Drafting of the manuscript: ET, FJA, IGC, JB, JIGH. Critical revision of the manuscript for important intellectual content: ET, FJA, IGC, JB, JIGH Administrative, technical, or material support: ET, FJA, IF, IGC, JB, JIGH. Study supervision: ET, SF, FJA, IGC, JB, JIGH.
